# Dermoscopic features of children scabies

**DOI:** 10.3389/fmed.2023.1097999

**Published:** 2023-02-21

**Authors:** Ying-li Nie, Hong Yi, Xiao-yan Xie, Gui-li Fu, Yuan-quan Zheng

**Affiliations:** Department of Dermatology, Wuhan Children’s Hospital (Wuhan Maternal and Child Healthcare Hospital), Tongji Medical College, Huazhong University of Science and Technology, Wuhan, China

**Keywords:** scabies, mite, dermatoscope, children, manifestations

## Abstract

Scabies is a common skin disorder, caused by the ectoparasite *Sarcoptes scabiei*. The scabies mites burrow is highly diagnostic but illegible by the naked eye, because it is tiny and may completely be obscured by scratch and crust. The classic technique is opening the end of an intact mite burrow with a sharp instrument and inspecting its contents in the light microscope under loupe vision. Dermatoscope is a new method to diagnose scabies, with the advantages of non-invasive and more sensitive. This study verified the characteristic manifestations of scabies under dermoscopy. Under the closer examination of the curvilinear scaly burrow, the scabies mite itself may be seen as a dark equilateral triangular structure, which is often referred to as a “jet with contrail.” Besides, this study found that the positive detection rate of microscopic characteristic manifestations under the dermoscopy ordered by the external genitals, the finger seams and the trunk, which were statistically different (*P*-value < 0.05). Of note, this is the first study to explore the regional distribution of the characteristic dermoscopic manifestations of scabies. We are the first to propose to focus on examining the external genitalia and finger seams with dermoscopy.

## Introduction

Scabies is a parasitic infestation of the skin caused by the mite *Sarcoptes scabiei*. Clinically, it is characterized by erythematous papules or vesicles. The typical distribution of skin lesions includes finger seams, the wrists, armpits, groins, buttocks, genitals and the breasts in women. In infants and young children, the palms, soles, and head are also commonly involved, which is rare in adults (1). Pruritus is the predominant hallmark of scabies regardless of age (2). Patients mainly complain the itchy rash worsening at night, which seriously disturbs sleep and affects the quality of life (3). Besides, the disease also causes high psychosocial and economic burden (4).

Burrows are formed as the adult female scabies mites excavate their way through the epidermis (5). The burrow is highly diagnostic. However, they are often unidentifiable by the naked eye, because scratch, crust and eczematization may completely obscure primary lesions (6). The only proof of the diagnosis of scabies is demonstration of scabies mite, its eggs or feces pellets (7). There are a variety of methods to diagnose scabies, but no one is both convenient and reliable. Therefore, the diagnosis of scabies is currently challenging and often delayed.

Dermatoscope is one of the new methods to diagnose scabies (8). As we know, dermatoscope allows better visualization on skin lesions, with the advantages of non-invasive, real-time observation and more sensitive mode of evaluation. There are few studies on dermoscopy in scabies, let alone children scabies. The current literatures mainly consist of case reports (9). In this article, we aim to analyze the dermoscopic features of children scabies.

## Materials and methods

### Patients selection and data collection

We performed a retrospective analysis of children patients with clinical scabies or suspected scabies in department of dermatology, Wuhan Children’s Hospital (Wuhan Maternal and Child Healthcare Hospital), from August 2020 to October 2022. All the included patients met the 2018 The International Alliance for the Control of Scabies (IACS) criteria for the diagnosis of scabies (10). The definition of the clinical scabies and suspected scabies was shown in Table 1. Those who had papular urticaria, atopic dermatitis, lichen planus, dermatitis herpetiformis, and infantile acropustulosis were excluded. The data of enrolled patients were collected by electronic medical records system, including demographic information, medical history, clinical symptoms and signs.

**TABLE 1:** Summary of the 2018 IACS criteria for the diagnosis of scabies.

A: Confirmed scabies	
At least one of:	A1: Mites, eggs or feces on light microscopy of skin samples A2: Mites, eggs or feces visualized on individual using high-powered imaging device A3: Mite visualized on individual using dermoscopy
**B: Clinical scabies**	
At least one of:	B1: Scabies burrows B2: Typical lesions affecting male genitalia B3: Typical lesions in a typical distribution and two history features
**C: Suspected scabies**	
One of:	C1: Typical lesions in a typical distribution and one history feature C2: Atypical lesions or atypical distribution and two history features
**History features**	
	H1: Itch H2: Close contact with an individual who has itch or typical lesions in a typical distribution

### Dermoscopy

The CBS dermoscopy detection system was used to scan the clinically suspicious skin lesions of the patients, including the finger seams, armpits, trunk, buttocks and genitalia, etc. The 50× lens was used to move back and forth on all clinical prone sites one by one. All dermoscopic pictures were saved from the computer after examination.

The most arresting feature of scabies under dermoscopy was the burrow. Dermoscopy began with looking for the scabies burrow, which was the movement path of the scabies mite in the stratum corneum of the patients. The burrow entrance was usually where the scales was visible (the start of the sarcoptic curve), and the end of the burrow was usually where the blister was located (the end of the curve).

Under the closer examination of the curvilinear scaly burrow, the scabies mite itself was usually located at the end of the burrow a few millimeters in front of the small blister. The end of the burrow was wiped with an alcohol cotton swab. Through the cuticles, the mite itself was seen at the end of the burrow as a dark equilateral triangular structure, corresponding to the pigmented head and anterior legs of the scabies mite. The burrow and the dark triangular structure were often referred to as a “jet with contrail” (11). Because the scabies mite was white and difficult to find, the target of dermoscopic observation was mainly the burrow and dark triangular structure.

### Statistical analysis

Continuous variables were displayed as means and standard deviation (SD). Categorical variables were expressed as frequency counts and percentages, and chi-square was used to evaluate the differences between groups. All statistics were analysed by using the SPSS 22.0 software, and *P*-value < 0.05 was selected as the threshold of statistical significance.

## Results

### Demographic features

We collected a total of 56 cases with clinical scabies and suspected scabies. All of them received dermoscopy examination. Among them, 34 patients were male, accounting for 60.7%, and 22 patients were female, accounting for 39.3%. The eldest patient was 16 years old and the youngest patient was an 8-month-old infant. Their mean (SD) age was 6.14 ± 4.07 years old.

### The characteristic dermoscopic manifestations of scabies

**FIGURE 1:**
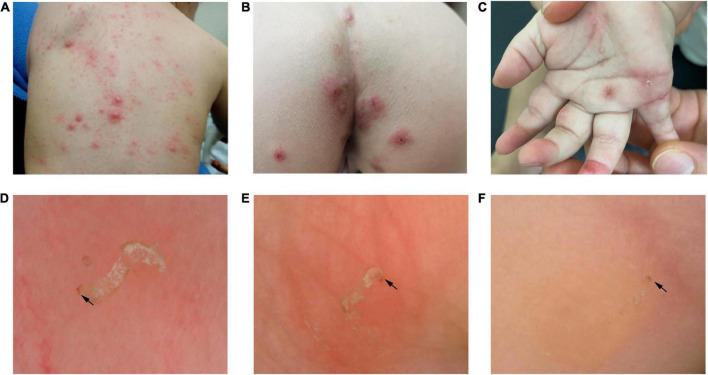
Panels **(A–C)** were clinical images. At the end of the curvilinear scaly burrow, the mite itself was seen as a dark triangular structure (black arrow) under dermoscope. Panel **(D)** was the dermoscopic image of trunk. Panel **(E)** was the dermoscopic image of external genital. Panel **(F)** was the dermoscopic image of finger seam.

**FIGURE 2:**
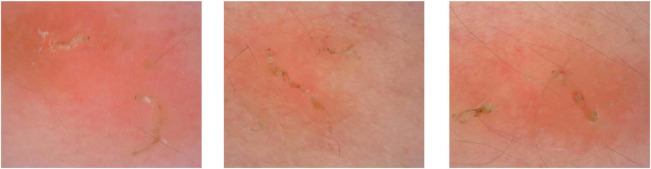
Multiple burrows were seen in one dermoscopic image.

In total, 50 patients presented the typical “jet with contrail” under dermoscopy. Those 50 cases were confirmed scabies. The burrow and dark triangular structure were shown on Figure 1. Even more, multiple burrows and dark triangular structure were seen in one dermoscopic image (Figure 2).

### The regional distribution of the characteristic dermoscopic manifestations of scabies

Due to the small amounts, in this study, the neck, chest wall, abdomen, back, armpits and buttocks were all divided into the trunk. Only 6 patients could be found that the burrow and the dark triangular structure were on the trunk, versus 41 on the finger seams and 45 on the external genitals, relatively. The detection rates of characteristic dermoscopic manifestations of the finger seams and the external genitals were statistically significant compared with those of the trunk (all *P*-value < 0.05). The regional distribution of the characteristic dermoscopic manifestations of confirmed scabies was shown in Table 2.

**TABLE 2:** The regional distribution of the characteristic dermoscopic manifestations of 50 confirmed scabies patients.

Area	Burrow under dermatoscope		χ*^2^*	*P*
	**Positive (%)**	**Negative (%)**		
Trunk	6 (12.0)	44 (88.0)		
Finger seams	41 (82.0)	9 (18.0)	49.177	<0.001*
External genitals	45 (90.0)	5 (10.0)	60.864	<0.001**

*Finger seams compared with trunk.

**External genitals compared with trunk.

### The non-characteristic dermoscopic manifestations of scabies

**FIGURE 3:**
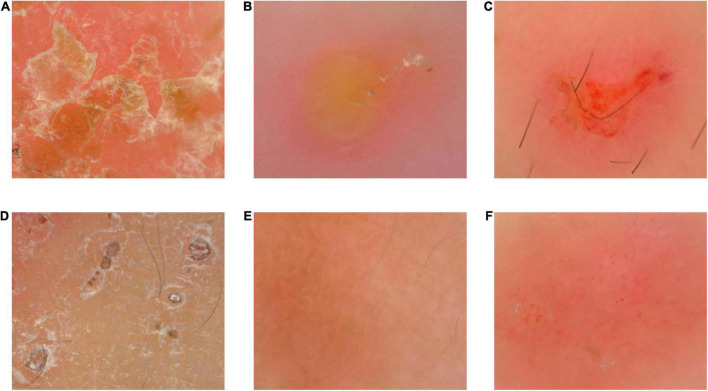
The non-specific dermoscopic manifestations of scabies. Panel **(A)** was erythema overlying scales. Panel **(B)** was pustule. The burrow and dark triangular structure were seen around the edge of pustule. Panel **(C)** was erosion and exudation. Panel **(D)** was scratches and blood callus. Panel **(E)** was pigmentation. Panel **(F)** was vascular changes. Punctate and globular vessels were focally distributed.

The non-specific dermoscopic manifestations of scabies were mainly secondary skin lesions caused by pruritus and local eczema changes, such as erythema overlying scales, pustules, erosion, exudation, scratches, blood callus, pigmentation, and vascular changes. They were shown in Figure 3.

## Discussion

Scabies can be both one of the easiest and one of the most difficult conditions to diagnose (6). The parasitological confirmation of the scabies mite forms the gold standard for diagnosis, including of the mite, its eggs or feces. The classic skin scrapings is by opening the end of an intact mite burrow with a sharp instrument and inspecting its contents in the light microscope under loupe vision. This test is poorly tolerated in children because of the pain and repeated tests from different sites may be needed (12). Adhesive tape test and burrow ink test (BIT) are painless and simple, but its sensitivity is low (13, 14). Reflectance confocal microscopy (RCM) and optical coherence tomography (OCT) are new non-invasive technique. They enable visualization of burrows, mite, larvae, eggs and fecal material (15, 16). The limitation of RCM and OCT is non-availability and expensive equipment. Histopathological examination can also allow visualization of burrows, female mite, eggs containing larvae, eggshells and fecal deposits (scyballa) (17). Nevertheless, skin biopsy is both time consuming and expensive.

Dermoscopy can be used to confirm the diagnosis of scabies in both adults and children, according to the IACS criteria (10). Nowadays, the “jet with contrail” was considered pathognomonic for scabies, regardless of the patient’s age, the location of the lesion, type and duration of the infect ion (18). Even if only one skin lesion on the whole body presented this typical dermoscopic manifestation, it was sufficient to confirm the diagnosis of scabies.

In this study, 56 patients received dermoscopy examination and 50 patients were confirmed the diagnosis of scabies by the characteristic “jet with contrail.” The sensitivity of dermatoscopic diagnosis of scabies was 89.3%, which was similar to what the literature has reported (19). There were six patients without characteristic dermoscopic features of the lesions throughout the body. The reason may be that erosion, scratches and blood callus covered and obscured primary lesions. Those six patients can be diagnosed with clinical scabies on account of the positive response to empiric treatment.

Because pruritus is so constant and violent in scabies, the scratching may catch viable mites, which can survive under the nails and then colonize the skin starting from around the nail going proximally (20). Scabies mites are more readily to crawling in areas where the epidermis is thin and tender. This may be the reason why skin lesions tend to occur between the finger seams, the wrists, armpits, groins, buttocks, and genitals.

This study found that the positive detection rate of microscopic structures under the dermoscopy ordered by the external genitals, the finger seams and the trunk, which were statistically different (*P*-value < 0.05). Its high detection rate may stem from the fact that the epidermis thickness of finger seams and external genitals was thinner and the transmission of light was better. Therefore, skin can easily expose the scabies mite. In addition, genital area was more private with less scratching, resulting in less damagement of the primary skin lesions, so the burrows were more unbroken.

It suggests that when we use dermoscopy in clinical examination of patients suspected of scabies, we should focus on observing the skin lesions of external genitalia and finger seams, which increased the credibility of dermoscopy and avoid missed diagnosis. Of note, this is the first study to explore the regional distribution of the characteristic dermoscopic manifestations of scabies. We are the first to propose to focus on examining the external genitalia and finger seams with dermoscopy in the diagnosis of scabies, which has great guiding value for clinical practice.

This study revealed the characteristic manifestations of scabies under dermoscopy. It clearly showed the parasitic state of scabies, and had a more intuitive understanding of scabies. Dermoscopy can effectively decrease the misdiagnosis rate of clinical physicians. Besides, this study showed the non-characteristic manifestations under dermoscopy, which was neglected in the past studies. The significance of non-specific structures was to indicate the complicated infection and allergic status of different patients, and to indicate the skin barrier damage of different degrees in scabies patients (21).

This study has limitations due to its retrospective design. And the relatively small size of sample is another limitation of our study. We look forward to multi-center, large sample prospective studies to enhance the confidence of the results. Furthermore, the limitations of dermoscopy are that eggs or fecal material are not visible (8). It can be challenging in hair-bearing areas and awkward to perform in genital or other sensitive areas. Besides, the device needs to be carefully disinfected because mites can survive in the environment up to 72 h (22).

## Data availability statement

The raw data supporting the conclusions of this article will be made available by the authors, without undue reservation.

## Author contributions

Y-LN and HY: design, analysis, and interpretation of data, and drafting of the manuscript. X-YX: acquisition of data and statistical analysis. G-LF and Y-QZ: critical revision of the manuscript for important intellectual content, obtaining funding, and supervision. All authors read and approved the final manuscript.
